# Genome sequence and methylome of the extremely halophilic bacterium *Salinibacter ruber* strain M31^T^ isolated from a crystallizer pond in Mallorca, Spain

**DOI:** 10.1128/mra.00856-25

**Published:** 2025-10-10

**Authors:** Beatriz Toledo Akiti, Gülay Kaya, Sean P. Kennedy, Priya DasSarma, Tamas Vincze, Alexey Fomenkov, Richard J. Roberts, Shiladitya DasSarma

**Affiliations:** 1 Institute of Marine and Environmental Technology, University System of Maryland 145781, Baltimore, Maryland, USA; 2 Blue Marble Space Institute of Science 824783, Seattle, Washington, USA; 3 Computational Biology Department, Institut Pasteur, Université Paris Cité 555089, Paris, France; 4 Department of Microbiology and Immunology, School of Medicine, University of Maryland 200790, Baltimore, Maryland, USA; 5 New England Biolabs 1696 , Ipswich, Massachusetts, USA; Portland State University, Portland, Oregon, USA

**Keywords:** extreme halophile, extremophile, bacteria, DNA methylation, horizontal gene transfer, rhodopsin, transposase

## Abstract

*Salinibacter ruber* strain M31^T^, an extremely halophilic bacterium, was isolated from a saltern crystallizer pond in Spain. Single-molecule real-time sequencing revealed a 3.6-Mbp genome with a single 3.55-Mbp circular chromosome and a 35.5-kbp plasmid. The highly acidic proteome includes a total of 2,962 proteins, some of which are archaeal-like.

## ANNOUNCEMENT

*Salinibacter ruber* is an extremely halophilic bacterium that represents an interesting model for convergent evolution with halophilic Archaea as well as astrobiological studies (1–3). Besides an elevated intracellular concentration of KCl for osmotic balance, it also possesses rhodopsins for phototrophic energy production and displays a high degree of genome plasticity (4). The gram-negative *Salinibacter ruber* strain M31^T^ was isolated from a saltern crystallizer pond in Mallorca, Balearic Islands, Spain (GPS: 39.3499° N, 3.0110° E) on September 1999 and was a gift from the American Type Culture Collection (1). *Salinibacter ruber* M31^T^ cultures were grown in ATCC 2402 medium (5). High-molecular weight genomic DNA was prepared by lysis and extraction with phenol:chloroform, followed by ethanol precipitation as described earlier (6).

DNA samples (~2 µg) were sheared to an average size of ~10 kb using the G-tube protocol (Covaris, MA). DNA libraries were prepared using a SMRTbell Express Template Prep Kit 2.0 (100-938-900, Pacific Biosciences, CA) and ligated with barcoded hairpin lbc--lbc adapters for multiplex sequencing on a Sequel II (SQ2) instrument. Incompletely formed SMRTbell templates were removed by digestion with a combination of exonucleases III and VII according to the manufacturer’s instructions (NEB, MA). The qualification and quantification of the SMRTbell libraries were made on a Qubit fluorometer (Invitrogen, OR) and a 2100 bioanalyzer (Agilent Technologies, CA). Size-selected separation of SMRTbell libraries on the gel-based BluePippin instrument (Sage Science, Beverly, MA) was performed to remove impurities and improve genome assembly results. Single-molecule real-time (SMRT) sequencing was performed on a PacBio Sequel II platform (Pacific Biosciences, CA) with a 2 h pre-extension and 30 h collection time. Sequencing reads were assembled *de novo* using a microbial genome annotation analysis pipeline (SMRT Link v11.1.0.166339) with default parameters. The 60,343 HiFi (CCS) reads with a 6,165 bp mean read length and 6,767-bp N50 read length yielded 374 Mb of high-quality sequencing data. The polished assembly resolved automatically into a large circular chromosome and circular plasmid pSR35 (Table 1).

**TABLE 1:** Genome properties of *Salinibacter rube*r M31^T^

Replicon properties	Chromosome	pSR35	Whole genome
Size (bp)	3,551,259	35,506	3,586,765
GC content (%)	66.2	57.9	66.1
Coverage	104	147	104
Gene no.	3,015	29	3,044
Encoded proteins no.	2934	28	2962
GenBank	CP169425	CP169426	GCF_041941415.1

**Fig 1:**
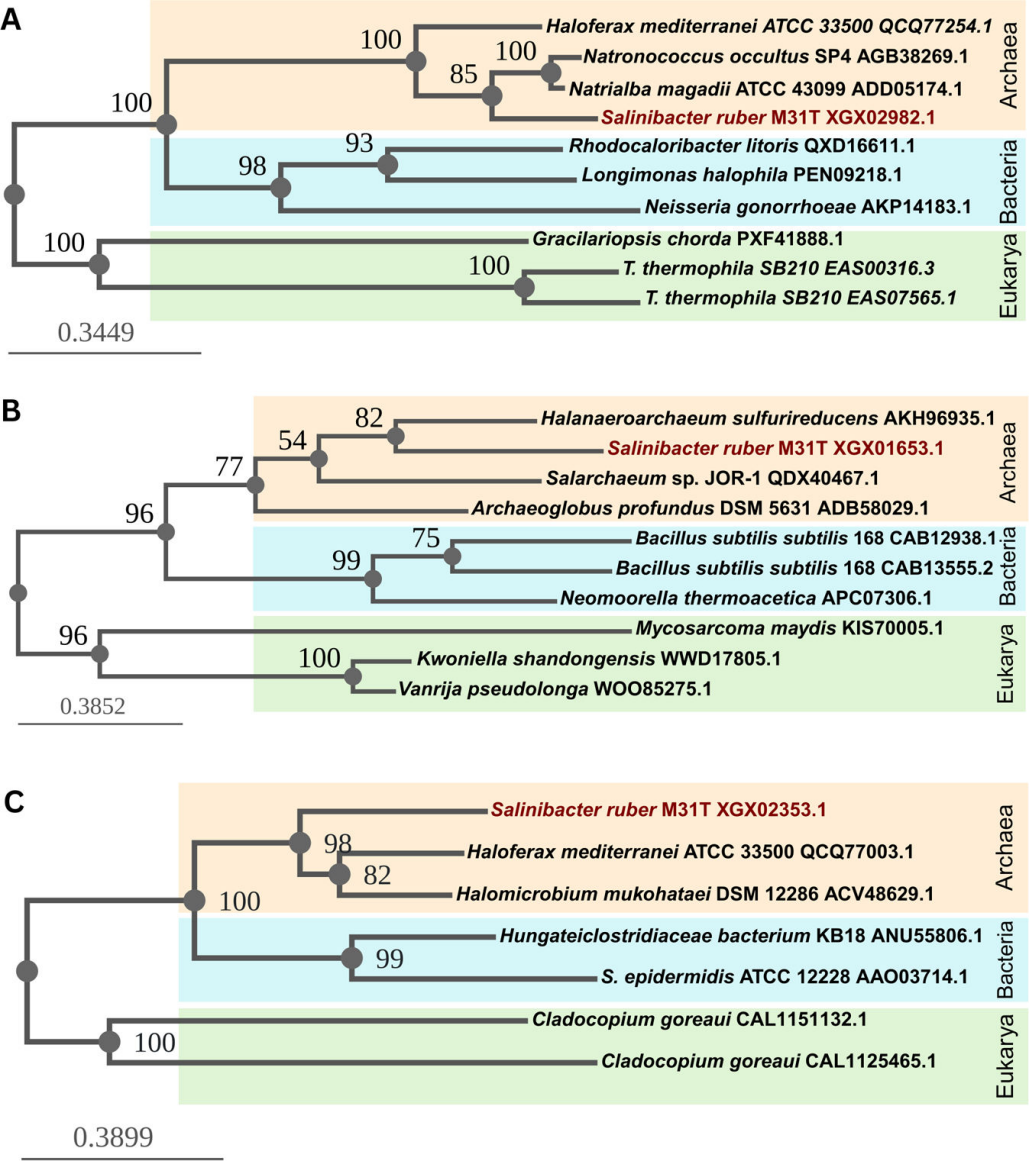
Maximum likelihood phylogenetic trees of the *S. ruber* M31^T^-predicted proteins with an archaeal evolutionary history potentially acquired by horizontal gene transfer: (**A**) sodium-dependent transporter (XGX02982.1); (**B**) MFS transporter (XGX01653.1); and (**C**) ABC transporter ATP-binding protein (XGX02353.1). The organism names are followed by the PID of the sequence from NCBI. The *S. ruber* M31^T^ sequences group with the archaeal sequences (orange) instead of bacterial (blue) or eukaryotic (green) ones.

Genome annotation was performed using GeneMark.hmm2, Prokka, and National Center for Biotechnology Information (NCBI) Prokaryotic Genome Annotation Pipeline build 3190 (7–9). The mean isoelectric point (pI) of 5.61 calculated using IPC 2.0 indicates an acidic proteome which, together with the GC-rich genome (Table 1), is characteristic of extreme halophiles (10–12). The genome includes a single rRNA operon and 44 tRNA genes predicted using tRNAscan-SE 2.0 (13). Based on analysis using BLAST, NCBI’s annotation and HaloWeb, two archaeal-like sensory rhodopsins I, a halorhodopsin, a xanthorhodopsin, four photolyases, as well as 50 annotated transposases—only one in pSR35—are located in its genome (14–16). Consistent with adaptation to their evaporitic environment, stress and heavy metal proteins are also predicted. Additionally, phylogenetic analysis using BLAST and Phylogeny.fr’s advanced workflow identified possible candidates for archaeal lateral transfer origin (Fig. 1) (14, 17).

Methylation patterns were determined by the same microbial genome analysis pipeline (SMRT Link v11.1.0.166339), yielding two m6A methylated DNA motifs: GAT**A**NNNNNC**T**C and CAGC**A**G. Homology-based searches of the assembled genome identified six restriction-modification (R-M) system genes that were recorded in the REBASE database (18). BLAST analysis was used to identify RM system genes from the complete genome sequence by using each open reading frame in the genome to query NCBI nr and the REBASE databases (18, 19). Multiple sequence alignments were performed using the PROMALS 3D server (20). Type I methylase M.SruM31TI (ACED82_05905) was assigned to the GAT**A**NNNNNC**T**C motif, and no gene was assigned to the CAGC**A**G motif yet.

## Data Availability

The *Salinibacter ruber* M31^T^ (BioProject PRJNA412908, BioSample SAMN43291986) whole-genome sequence was deposited at GenBank under accession numbers CP169425 (chromosome) and CP169426 (pSR35). The raw data are available in the NCBI Sequence Read Archive (SRR30573099).
